# Supplementary material for: Developmental transcriptomics of the Firebrat: Exploring developmental expression patterns and morphology during the embryogenesis of Thermobia domestica
Source: PLoS One. 2025 Jun 5;20(6):e0324844. doi: 10.1371/journal.pone.0324844 (PMC12140273; doi:10.1371/journal.pone.0324844)
Supplement: S1 Table — Information on the dates and number of eggs sampled for each sample of the temporal developmental expression atlas study. Sample names ending with numbers were pooled. ELP, egg-laying period; hAEL, hours after egg-laying. (PDF) [file pone.0324844.s001.pdf]

Sample	*n eggs*	*Start ELP*	*End ELP*	*Sampling Date*
9hAEL_A.1	89	25/10/2021 22:00	26/10/2021 07:00	26/10/2021 07:00
9hAEL_A.2	74	27/10/2021 22:00	28/10/2021 07:00	28/10/2021 07:00
9hAEL_B.1	88	26/10/2021 22:00	27/10/2021 07:00	27/10/2021 07:00
9hAEL_B.2	66	28/10/2021 22:00	28/10/2021 07:00	29/10/2021 07:00
9hAEL_C	214	09/11/2021 22:00	10/11/2021 07:00	10/11/2021 07:00
16hAEL_A.1	107	02/11/2021 22:00	03/11/2021 07:00	03/11/2021 14:00
16hAEL_A.2	42	10/11/2021 22:00	11/11/2021 07:00	11/11/2021 14:00
16hAEL_B	117	03/11/2021 22:00	04/11/2021 07:00	04/11/2021 14:00
16hAEL_C	250	10/11/2021 22:00	11/11/2021 07:00	11/11/2022 14:00
24hAEL_A.1	70	16/11/2021 07:30	16/11/2021 16:30	17/11/2021 07:30
24hAEL_A.2	30	22/11/2021 07:45	22/11/2021 16:45	23/11/2021 07:45
24hAEL_B	134	02/11/2021 07:00	02/11/2021 16:00	03/11/2021 07:00
24hAEL_C	214	03/11/2021 07:00	03/11/2021 16:00	04/11/2021 07:00
36hAEL_A	59	22/11/2021 22:00	23/11/2021 07:00	24/11/2021 22:00
36hAEL_B	60	29/11/2021 22:00	30/11/2021 07:00	01/12/2021 22:00
36hAEL_C	170	10/11/2021 22:00	11/11/2021 07:00	12/11/2021 22:00
48hAEL_A.1	30	15/11/2021 08:00	15/11/2021 17:00	17/11/2021 08:00
48hAEL_A.2	20	22/11/2021 07:45	22/11/2021 16:45	24/11/2021 07:45
48hAEL_B	53	15/11/2021 08:00	15/11/2021 17:00	17/11/2021 08:00
48hAEL_C	151	15/11/2021 08:00	15/11/2021 17:00	17/11/2021 08:00
60hAEL_A	90	15/11/2021 22:00	16/11/2021 07:00	18/11/2021 10:00
60hAEL_B	90	15/11/2021 22:00	16/11/2021 07:00	18/11/2021 10:00
60hAEL_C	110	15/11/2021 22:00	16/11/2021 07:00	18/11/2021 10:00
72hAEL_A	39	19/11/2021 09:00	19/11/2021 18:00	22/11/2021 09:00
72hAEL_B	38	19/11/2021 09:00	19/11/2021 18:00	22/11/2021 09:00
72hAEL_C	69	19/11/2021 09:00	19/11/2021 18:00	22/11/2021 09:00
96hAEL_A	30	11/11/2021 07:15	11/11/2021 16:15	15/11/2021 07:15
96hAEL_B	31	11/11/2021 07:15	11/11/2021 16:15	15/11/2021 07:15
96hAEL_C	50	11/11/2021 07:15	11/11/2021 16:15	15/11/2021 07:15
120hAEL_A	30	17/11/2021 07:30	17/11/2021 16:30	22/11/2021 07:30
120hAEL_B	30	17/11/2021 07:30	17/11/2021 16:30	22/11/2021 07:30
120hAEL_C	50	17/11/2021 07:30	17/11/2021 16:30	22/11/2021 07:30
144hAEL_A	30	12/11/2021 09:00	12/11/2021 18:00	17/11/2021 08:00
144hAEL_B	27	12/11/2021 09:00	12/11/2021 18:00	17/11/2021 08:00
144hAEL_C	35	12/11/2021 09:00	12/11/2021 18:00	17/11/2021 08:00
168hAEL_A	40	12/11/2021 09:00	12/11/2021 09:00	19/11/2021 09:00
168hAEL_B	40	12/11/2021 09:00	12/11/2021 09:00	19/11/2021 09:00
168hAEL_C	64	12/11/2021 09:00	12/11/2021 09:00	19/11/2021 09:00
192hAEL_A	30	10/11/2021 07:30	10/11/2021 16:30	18/11/2021 07:30
192hAEL_B	30	10/11/2021 07:30	10/11/2021 16:30	18/11/2021 07:30
192hAEL_C	37	10/11/2021 07:30	10/11/2021 16:30	18/11/2021 07:30
216hAEL_A	21	09/11/2021 09:30	09/11/2021 18:30	18/11/2021 09:30
216hAEL_B	21	09/11/2021 09:30	09/11/2021 18:30	18/11/2021 09:30
216hAEL_C	36	09/11/2021 09:30	09/11/2021 18:30	18/11/2021 09:30
240hAEL_A	30	08/11/2021 20:45	08/11/2021 17:45	18/11/2021 08:45
240hAEL_B	30	08/11/2021 20:45	08/11/2021 17:45	18/11/2021 08:45
240hAEL_C	36	08/11/2021 20:45	08/11/2021 17:45	18/11/2021 08:45

Table S1. Thermobia domestica egg sampling overview.
Information on the dates and number of eggs sampled for each sample of the temporal developmental expression atlas study. Sample names ending with numbers were pooled. ELP, egg laying period; hAEL, hours after egg laying.
