# Supplementary material for: Developmental transcriptomics of the Firebrat: Exploring developmental expression patterns and morphology during the embryogenesis of Thermobia domestica
Source: PLoS One. 2025 Jun 5;20(6):e0324844. doi: 10.1371/journal.pone.0324844 (PMC12140273; doi:10.1371/journal.pone.0324844)
Supplement: S1 Fig — Expression data throughout development in hours after egg-laying (hAEL) is presented in log2-centered TMM (+1) values. Mean expression values are indicated in black. (PDF) [file pone.0324844.s002.pdf]

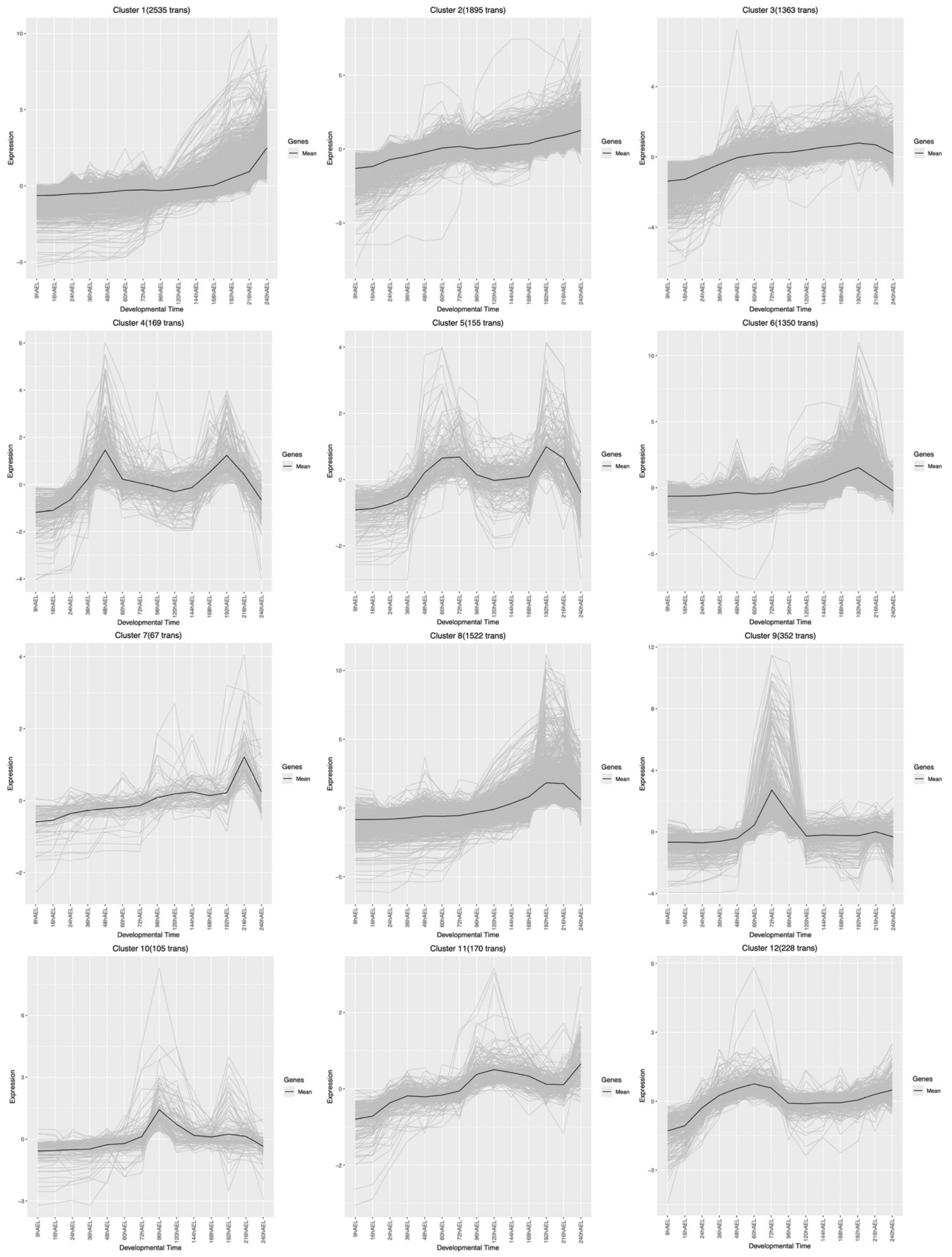

Figure S1. Expression levels of transcripts belonging to clusters 1-12.
Expression data throughout development in hours after egg laying (hAEL) is presented in log2-centered TMM (+1) values. Mean expression values are indicated in black.
